# Effects of oral supplementation of β -hydroxy-β -methylbutyrate on muscle mass and strength in individuals over the age of 50: a meta-analysis

**DOI:** 10.3389/fnut.2025.1522287

**Published:** 2025-04-03

**Authors:** Nan Li, Shanbin Chen, Yuxi He, Yamin Chen, Xiaosa Duan, Wenxing He, Qihe Wang, Sana Liu, Tongbo Liu, Haiqin Fang

**Affiliations:** ^1^NHC Key Laboratory of Food Safety Assessment, Chinese Academy of Medical Science Research Unit (2019RU014), China National Center for Food Safety Risk Assessment, Beijing, China; ^2^School of Biological Science and Technology, University of Jinan, Jinan, China; ^3^Institute of Food and Nutrition Science and Technology, Shandong Academy of Agricultural Sciences, Jinan, China; ^4^Northwest Agriculture and Forestry University, Yangling, Shanxi, China; ^5^Department of Information, Medical Supplies Center of PLA General Hospital, Beijing, China

**Keywords:** β-Hydroxy-β-methylbutyrate, dosage, duration, muscle, mass, strength, function, metaanalysis

## Abstract

**Background:**

β-Hydroxy β-Methylbutyrate (HMB) has shown potential in improving muscle protein turnover, which may be important for preventing muscle degradation in aging populations. The aim of this meta-analysis is to clarify the impact of HMB oral supplementation on muscle-related indicators and discover the interaction between dosage and duration. The findings will provide a scientific basis for the use of HMB oral supplementation in the management of muscle attenuation.

**Methods:**

A computer systems-based search for articles of randomized controlled trial (RCT) in the PubMed, Cochrane Library, Web of Science, ScienceDirect, EBSCO English databases and China Journal Full-Text Database (CNKI), Wan Fang Chinese databases, was conducted up to October 2023. Data were pooled using weighted mean difference (WMD) and 95% confidence intervals (95% CI).

**Results:**

A total of 21 RCTs were included, involving 1935 participants, all of them were > 50 years old. Results showed a positive impact of HMB oral supplementation in improving muscle mass (appendicular skeletal muscle mass: WMD = 1.56 kg, 95% CI: 0.03–3.09 kg and lean mass: WMD = 0.28 kg, 95% CI: 0.16–0.41 kg), strength (handgrip strength: WMD = 0.54 kg, 95% CI: 0.04–1.04 kg and five-time chair stand test: WMD = –0.73 s, 95% CI: –1.35, –0.11 s), and physical function (gait speed: WMD = 0.06 m/s, 95% CI: 0.01–0.10 m/s). Subgroup analysis revealed that the effect of a dosage of 3 g/d had significant improvement, and the effect of supplementation duration lasting > 12 weeks had significant improvement, and a dosage of 3g/day for more than 12 weeks was recommended.

**Conclusion:**

HMB oral supplementation can improve muscle mass, strength, and physical function. We recommend to implement supplementation at a dosage of 3 g for a duration exceeding 12 weeks to achieve optimal benefits.

**Systematic review registration:**

https://www.crd.york.ac.uk/PROSPERO/login, identifier 42024518958.

## Introduction

Muscle acts as the body’s second heart, is crucial for bone health, consumes blood glucose, and is vital in human metabolism. Middle-aged people lose 3% of their muscle strength and 1% of their muscle mass each year on average (1). Skeletal muscle loss is about 30% at the age of 60 years and about 50% at the age of 80 years (1). Muscle attenuation is usually associated with imbalance between muscle catabolism and anabolism. With increasing age, the body resists normal growth signals, resulting in increased catabolism, which in turn leads to muscle loss and sarcopenia (2). Sarcopenia is a disease characterized by the decline in muscle mass, strength, and function with age (3). This condition may have detrimental effects on the health of older individuals, including an increased risk of falls, fractures, obesity, hypertension, diabetes, and cardiovascular diseases (4–6). The prevalence of sarcopenia in the elderly ≥ 65 years has been reported to be 20% in the Western population, and it reaches 50–60% in those aged ≥ 80 years (7). China has the largest elderly population, and the prevalence of sarcopenia among the community-dwelling elderly is also as high as 12% (8). Growing evidence suggests that sarcopenia is associated with adverse consequences and an increased medical burden (9). It can serve as a predictor for infection risk, length of hospital stay, readmission, hospital complications, decreased physical function, and mortality (10, 11). Consequently, there has been a growing interest in research on the prevention and treatment of sarcopenia in recent years.

HMB, an *in vivo* metabolite of leucine, has shown potential for improving muscle protein turnover. It is considered the most important regulator of muscle protein anabolism and has been found to promote muscle protein synthesis and inhibit muscle protein decomposition (12). Thus, HMB may be important for preventing muscle degradation in aging populations. Studies have demonstrated that HMB supplementation can improve muscle function, prevent muscle atrophy, promote wound healing, and enhance muscle mass and strength (12–14). Several studies have reported that HMB supplementation has a positive effect on muscle mass, strength, and physical function in the elderly (15–17). Considering the potential of HMB, understanding its role and optimizing its application in the treatment of sarcopenia are important. However, most previous studies have focused on elderly individuals > 65 years. Thus, there is a lack of research involving people aged between 50 and 65 years. After age 50, skeletal muscle mass and muscle strength gradually decrease; specifically, the leg muscle mass decreases by 1–2% per year and muscle strength decreases by 1.5–5% per year (18). Previous studies suggest that the long-term effect of supplementation is beneficial (19, 20). To ensure the safety of participants, the recommended supplement dosage is typically ≤ 3 g/day. However, studies on humans report no side effects with dosages as high as 6 g/day for up to 1 month (16, 21, 22). Thus, the specific role and dosage–duration response relationship of HMB in people > 50 years remain unclear. To provide clear dosage and duration guidance for HMB supplementation, a meta-analysis of the dosage–duration response relationship is warranted. This study focused on participants over the age of 50 and referred to the 2021 Chinese expert consensus on the diagnosis and treatment of sarcopenia in the elderly and the Asia Working Group for Sarcopenia (AWGS) consensus (23, 24) to identify the most clinically relevant evaluation indexes. We selected the appendicular skeletal muscle mass (ASMM) and lean mass (LM) as the evaluation indexes for muscle mass, handgrip strength and the five-time chair stand test as the evaluation indexes for muscle strength, and gait speed and the 6-min walk test (6MWT) as the evaluation indexes for physical function. Using meta-analysis, the effects of HMB oral supplementation on muscle mass, strength, and physical function were first analyzed. Furthermore, the effects of different dosage and duration of HMB oral supplementation on muscle-related indicators were explored. The optimal dosage-duration response relationship was obtained. The aim of the study was to provide evidence for the prevention and treatment of clinical sarcopenia.

## Materials and methods

This review was carried out in accordance with a protocol registered on PROSPERO (CRD: 42024518958). The method and results are reported according to the PRISMA (Preferred Reporting Items for Systematic Reviews and Meta-Analysis) guidelines (25) A compiled PRISMA checklist is included in Supplementary material.

### Literature search strategy

A computer systems-based search and sorting of relevant documents published before October 2023 was conducted in several databases, including PubMed, Cochrane Library, Web of Science, ScienceDirect, EBSCO, China Journal Full-Text Database (CNKI), and Wan Fang. In addition, references of included studies were retrieved for eligible studies. The search was conducted in both the Chinese and English languages. A free combination of subject words and free words was used in all searches (see Supplementary material for the search strategy).

### Literature inclusion criteria

The PICOS (population, interventions, comparators, outcomes, study design) criteria for the eligibility of studies (26) was used to determine the inclusion and exclusion criteria, as follows:

(1) Participants: The research participants should be adults > 50 years. We have no restrictions on participants’ gender, health status, socioeconomic status, race or geographical area.
(2) Intervention: Including at least a 6-week trial of any oral supplementation containing HMB. We included any HMB dosage, supplementation form (powders, pills, nutritional drink), and both intervention and the control groups were included in the study with the same exercise program.
(3) Comparators: Participants who did not receive HMB oral supplements (placebo, uniform hospital meals or standard diet).
(4) Outcomes: The study should be complete, and the outcome measures should include at least one of the following: ASMM, LM, handgrip strength, five-time chair stand test, gait speed, and 6MWT. The outcome indicators should be expressed using corresponding statistical indicators.
(5) Study designs: The studies should be RCTs. Study designs included double-blinded-randomized clinical trials. Randomization is mainly aided by randomization schedules, the electronic data capture system was utilized to assign participant numbers and randomized participants according to the generated randomization schedules. The blinded method was applied through the entire course of the study. Neither the investigators, staff involved in the study, or participants were informed of the identity of any of the study products over the entire study period. Studies not reporting randomization or blinding procedures were also included for evaluation, but this type of studies is considered high risk in the domain allocation concealment and blinding of participants and personnel.

### Literature screening and data extraction

Two researchers independently used EndNote version X9 (Clarivate Analytics, PA, United States) software for literature screening. In cases where their opinions differed, they discussed with a third researcher to make a decision on whether to include the literature. A standardized data extraction table was used to extract data, and cross-checking was conducted to identify any discrepancies in the data, which were subsequently corrected. Only baseline and endpoint of the intervention outcome data were retrieved if a study had multiple time points.

Data on general study characteristics, such as author name; publication year; nationality; intervention plan; study type; and basic information about the research participant, including age, sex, body mass index, and health status, were extracted. In addition, outcome indicators, including sample sizes of the intervention and control groups, as well as relevant data for the indicators used in this study, were also extracted. For studies with multiple intervention groups, we extracted data from each intervention group and the control group. The indicators were then classified, and the data were converted to obtain the available data.

### Quality evaluation

Two researchers used RevMan 5.3 (RevMan, V.5.3. Copenhagen: The Nordic Cochrane Centre, The Cochrane Collaboration, 2014) software to evaluate the quality of the studies based on the Cochrane bias risk assessment tool (Cochrane Handbook for Systematic Reviews of Interventions version 6.0) (27). The evaluation consisted of seven items: (1) whether the generation of random sequences was done correctly; (2) whether the random allocation scheme was concealed; (3) whether a blind method was implemented for both the research participants and the interveners; (4) whether the outcome evaluator was blinded; (5) whether the outcome indicators were complete; (6) whether there was selective reporting of study results; and (7) whether there were other sources of bias.

### GRADE assessment

The strength of the evidence presented in the study was evaluated using guidelines established by the Grading of Recommendations Assessment, Development and Evaluation (GRADE) Working Group. We classify the quality of evidence into four levels: very low, low, moderate, and high (28). The evaluation included five aspects: bias risk, inconsistency, indirectness, imprecision, and publication bias.

### Data analysis

The data analysis was performed using Stata version 12 (Statacorp LP, College Station, TX, United States) statistical software. The effect sizes were calculated with the random-effect model (DerSimonianLaird method) and were expressed as a weighted mean difference (WMD) with a 95% confidence interval (CI). Heterogeneity among the included studies was assessed using the I^2^ statistics. In line with the Cochrane Handbook for Systematic Reviews of Interventions, the interpretation threshold was set to 50% and 0.1. Funnel plots were used to assess the risk of bias, and Egger’s test was applied to asymmetry. It is known that health status, age, gender, BMI, frequency of supplementation, combination with exercise and combination with other substrates can all greatly affect muscle mass, strength, and physical function. In addition, dosage, duration, and relationship of dosage-duration are also factors affect the effectiveness of HMB intervention. So we performed subgroup analysis based on dosage (3 g/day and < 3 g/day), duration (> 12 weeks and ≤ 12 weeks), relationship of dosage-duration (3 g/day and > 12 weeks, 3g/day and ≤ 12 weeks, <3 g/day and > 12weeks, < 3g/day and ≤ 12 weeks), health status (diseased and healthy), age (< 65 years and ≥ 65 years), gender (male and female), BMI (< 25 kg/m^2^, ≥ 25 kg/m^2^), supplementary frequency (1 times/day, 2 times/day and 3 times/day), combination with exercise (yes and no) and combination with other substances (yes and no).

## Results

### Literature search results

**FIGURE 1:**
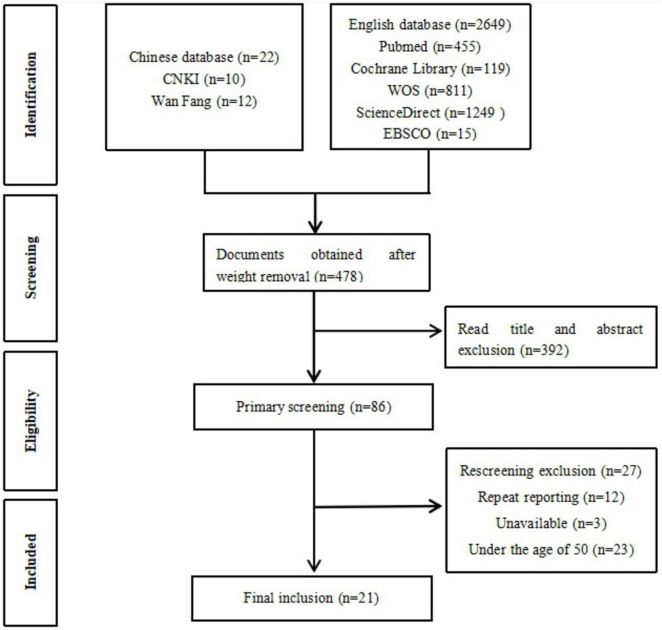
Flowchart illustrating various phases of the search and study selection according to the PRISMA (Preferred Reporting Items for Systematic Reviews and Meta-Analyses) guidelines.

Based on the search strategy, a total of 2,671 studies were initially identified. After screening, 21 RCTs (three in the Chinese language and 18 in the English language) were included in the analysis. The screening process is shown in Figure 1.

### Basic characteristics and quality evaluation of the included studies

**FIGURE 2:**
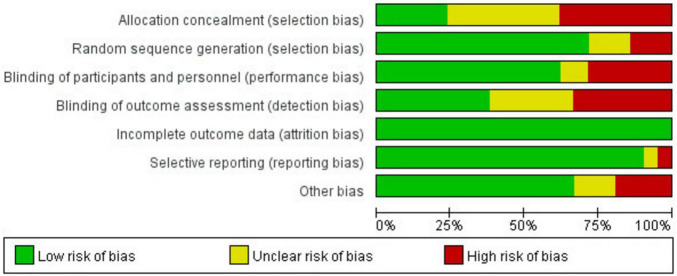
Risk-of-bias proportion for all studies.

**FIGURE 3:**
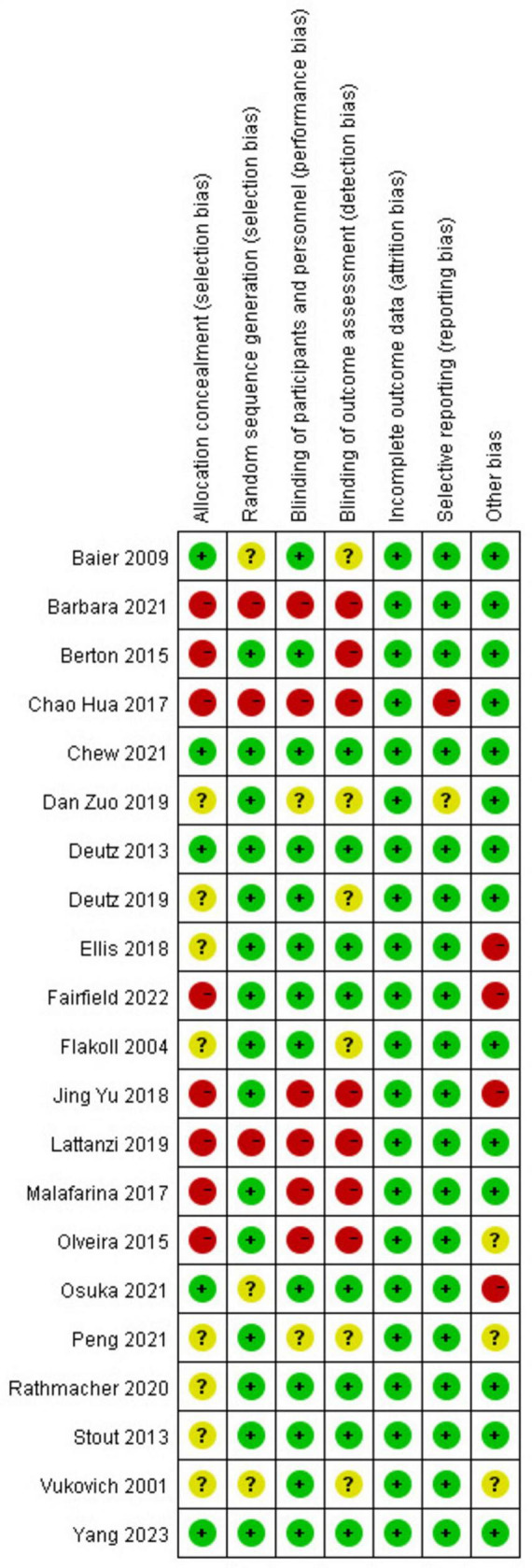
Risk-of-bias summary for all studies.

A total of 21 RCTs (29–49) were included in the meta-analysis. Among these studies, seven were conducted in Asia, six in Europe, and eight in America. The total sample size was 1935, with 967 cases in the experimental group and 968 cases in the control group. The basic characteristics of the included studies are summarized in Table 1. The quality of the included studies was evaluated, and the results (Figures 2, 3) indicated that the quality was high, making them suitable for meta-analysis.

**TABLE 1:** Study characteristics of included literatures.

Author, year	Country	Sample volume (intervention/control)	Intervention duration	Age		BMI (kg/m^2^)		State of health	Sex	Intervention group supplement	Control supplement	Frequency	Exercise plan	Dosage (g/d)	Outcome indicator
				**Intervention**	**Control**	**Intervention**	**Control**								
Zuo et al. (29)	China	25/23	6 weeks	70.20(4.29)	70.50(5.78)	23.14(5.19)	23.12(6.11)	Hip fracture patients	M/F	Standard diet+HMB	Standard diet	2	N	3	①③
Malafarina et al. (30)	Spain	36/38	7 weeks	85.7 (6.5)	84.7 (6.3)	24.9 (4.4)	26.0 (5.4)	Hip fracture patients	M/F	Standard diet+HMB	Standard diet	2	N	3.08	①②③⑤
Chew et al. (31)	Singapore	296/303	24 weeks	74.26(0.36)	74.04 (0.36)	18.36(0.09)	18.48(0.09)	Risk of Malnutrition	M/F	HMB	Placebo	2	N	1.48	①③
Hua et al. (32)	China	45/46	12 weeks	72.34(6.79)	72.34(6.79)	21.91(2.88)	21.91(2.88)	Risk of malnutrition	M/F	HMB	Placebo	2	N	2.6	①
Vukovich et al. (33)	America	14/17	8 weeks	70 (1)	70 (1)	–	–	Healthy	M/F	HMB	Placebo	3	N	3	②
Yu and Chai (34)	China	42/42	12 weeks	68.31(3.29)	68.28(3.19)	–	–	Lower extremity fracture and malnutrition patients	M/F	Uniform hospital meals+HMB	Uniform hospital meals	2	N	2.6	①
Deutz et.al. (35)	America	10/8	10 weeks	67.4 (1.4)	67.1 (1.7)	24.9 (1.0)	26.5 (1.2)	Healthy	M/F	CaHMB	Placebo	2	N	3	②
Stout et.al. (36)	America	49/49	24 weeks	73 (1)	73 (1)	26 (1)	25 (1)	Healthy	M/F	C_*a*_HMB	Placebo	2	Divided into two RCTs, the first without exercise. The second control group and the intervention group had the same resistance exercise.	3	②③
Rathmacher et.al. (37)	America	57/60	48 weeks	71.0 (1.1)	70.8 (1.1)	28.9 (1.0)	31.8 (0.9)	Insufficient, but not clinically deficient 25OH-D levels	M/F	C_*a*_HMB/Vitamin D_3_	Placebo	2	Divided into two RCTs, the first without exercise. The second control group and the intervention group had the same resistance exercise.	3	②
Baier et.al. (38)	America	40/37	48 weeks	75.41(1.53)	76.15(1.58)	–	–	Healthy	M/F	CaHMB L-Arginine/L-Lysine	Placebo	1	N	≤ 68 kg (2) > 68 kg (3)	②
Fairfield et.al. (39)	America	19/20	12 weeks	52 (1)	52.5 (1)	25.5 (2)	27.5 (1)	Healthy	F	C_*a*_HMB /Vitamin D_3_	Placebo	2	N	3	②
Ellis et.al. (40)	America	16/15	24 weeks	72.3 (6.6)	70.6 (5.2)	29.4 (4.5)	27.5 (4.0)	Healthy	M/F	HMB /Arginine/Glutamine	Placebo	2	N	3	②
Berton et.al. (41)	Italy	32/33	8 weeks	65-74	65-74	–	–	Healthy	F	C_*a*_HMB	Placebo	1	N	1.5	②③⑥
Olveira et.al. (42)	Spain	14/14	24 weeks	58.4(12.9)	53.7(13.1)	25.9 (3.4)	27.3 (5.8)	Patients with bronchiectasis	M/F	HMB	Placebo	1	Both groups performed the same exercise for 60 minutes per week.	1.5	②③
Flakoll et.al. (43)	America	41/36	12 weeks	77.7 (1.5)	75.7 (1.6)	–	–	Healthy	F	CaHMB/Arginine/Lysine	Placebo	1	N	2	②③
Osuka et.al. (44)	Japan	73/76	12 weeks	73.5 (4.2)	71.8 (4.1)	21.3 (2.2)	20.9 (2.1)	Low muscle mass	F	C_*a*_HMB	Placebo	1	N	1.5	②③④⑤
Yang et.al. (45)	China	18/18	12 weeks	72.89(7.02)	71.44(5.22)	–	–	Sarcopenia	M/F	C_*a*_HMB	Placebo	2	Both groups received the same resistance exercise.	3	②③④⑤
Peng et.al. (46)	China	29/33	12 weeks	70.66(4.16)	71.48(3.46)	22.43(3.57)	22.65(2.21)	Pre-frail older persons	M/F	HMB	Standard diet	2	N	3	④
Deutz 2019(47)	America	109/105	12 weeks	74.5 (7.3)	75.2 (7.6)	23.3 (5.4)	22.8 (5.0)	Patients with chronic obstructive pulmonary disease	M/F	HMB	Placebo	2	N	3	③
Lattanzi et.al. (48)	Italy	14/10	12 weeks	59.2 (8.4)	56 (4.6)	29.6(6.8)	29.8 (4.3)	Liver transplant recipients	M/F	HMB	Placebo	1	N	3	③④⑥
Lattanzi et.al. (49)	Italy	12/9	12 weeks	60.4 (5.4)	59.3 (7.3)	24.5 (3.0)	25.7 (4.9)	Patients with Liver Cirrhosis	M	HMB+fruit juice	fruit juice	2	N	3	③⑥

①ASMM; ②LM; ③Handgrip strength; ④Five-time chair stand test; ⑤Gait speed; ⑥6MWT; “-”No data.

### Meta-analysis results

#### Muscle mass

**FIGURE 4:**
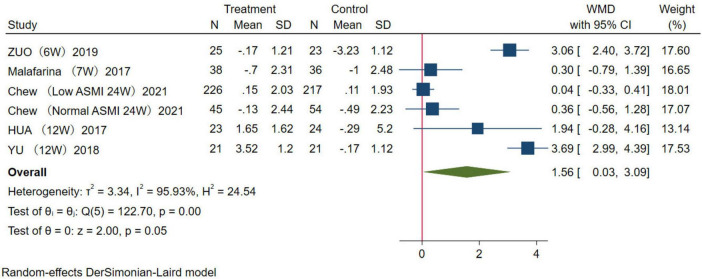
Forest plot of the effect of β-Hydroxy β-Methylbutyrate (HMB) on appendicular skeletal muscle mass (ASMM). The area of each square is proportional to the study’s weight in the meta-analysis. The vertical line and diamond indicate the overall measure of the effects and confidence intervals, respectively.

**FIGURE 5:**
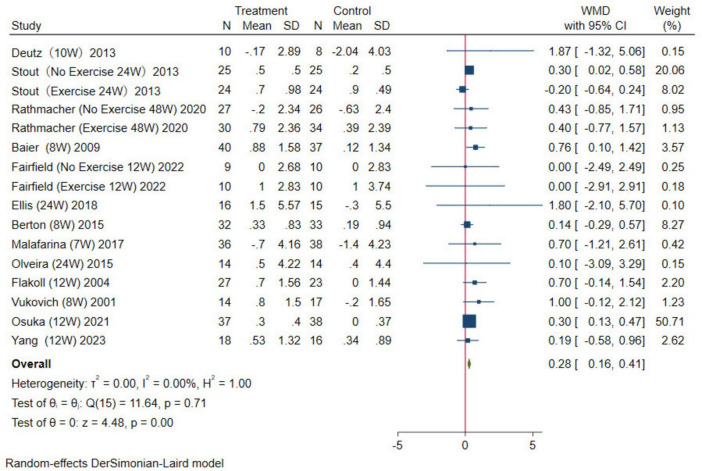
Forest plot of the effect of HMB on lean mass (LM). The area of each square is proportional to the study’s weight in the meta-analysis. The vertical line and diamond indicate the overall measure of the effects and confidence intervals, respectively.

In this study, two indicators, ASMM and LM, were used to evaluate muscle mass. Among the included studies, five reported the effect of HMB supplementation on ASMM. The results were analyzed using a random-effects model (WMD = 1.56 kg, 95% CI: 0.03–3.09 kg, *p* = 0.05; Figure 4). For LM, a total of 13 studies were combined, the results were analyzed using a random-effects model (WMD = 0.28 kg, 95% CI: 0.16–0.41 kg, *p* = 0.01; Figure 5). Overall, analysis of the two indicators revealed statistically significant differences between the HMB oral supplementation and control groups, indicating that HMB oral supplementation has a positive effect on ASMM and LM, particularly in individuals > 50 years.

#### Muscle strength

**FIGURE 6:**
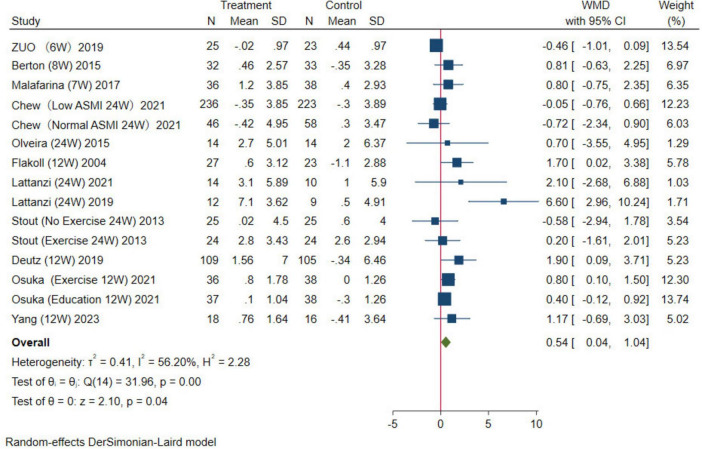
Forest plot of the effect of HMB on handgrip strength. The area of each square is proportional to the study’s weight in the meta-analysis. The vertical line and diamond indicate the overall measure of the effects and confidence intervals, respectively.

**FIGURE 7:**
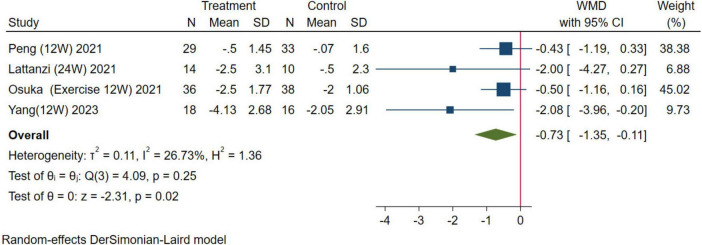
Forest plot of the effect of HMB on five-time chair stand test. The area of each square is proportional to the study’s weight in the meta-analysis. The vertical line and diamond indicate the overall measure of the effects and confidence intervals, respectively.

In this study, handgrip strength for upper limb muscle strength and the five-time chair stand test for lower extremity muscle strength were used as evaluation indexes for muscle strength. For handgrip strength, 12 studies were combined, the results were analyzed using a random-effects model (WMD = 0.54 kg, 95% CI: 0.04–1.04 kg, *p* = 0.04; Figure 6). For the five-time chair stand test, four studies were combined, the results were analyzed using the random-effects model (WMD = –0.73 s, 95% CI: –1.35, –0.11 s, *p* = 0.02; Figure 7). Overall, the results of both indicators demonstrated significant effects, indicating that HMB oral supplementation can increase handgrip strength and shorten the time required for the five-time chair stand test. This suggests that HMB oral supplementation can improve muscle strength in individuals > 50 years.

#### Physical function

**FIGURE 8:**
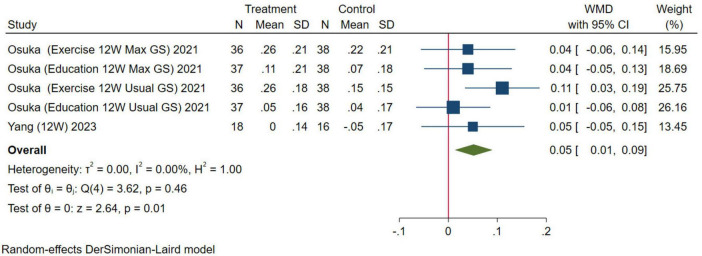
Forest plot of the effect of HMB on gait speed. The area of each square is proportional to the study’s weight in the meta-analysis. The vertical line and diamond indicate the overall measure of the effects and confidence intervals, respectively.

**FIGURE 9:**
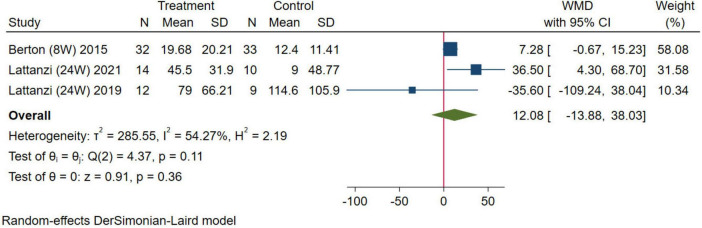
Forest plot of the effect of HMB on 6-min walk test (6MWT). The area of each square is proportional to the study’s weight in the meta-analysis. The vertical line and diamond indicate the overall measure of the effects and confidence intervals, respectively.

In this study, gait speed and the 6MWT were selected as indexes to evaluate physical function. For gait speed, five studies were combined, the results were analyzed using the random-effects model (WMD = 0.05 m/s, 95% CI: 0.01–0.09 m/s, *p* = 0.01; Figure 8). For the 6MWT, three studies were combined, the results were analyzed using the random-effects model (WMD = 12.08 m, 95% CI: –13.88–38.03 m, *p* = 0.36; Figure 9). The results of gait speed demonstrated statistically significant impact, indicating that HMB oral supplementation can improve gait speed. And the results of 6MWT are also positive, but due to the limitation in the number of literature, the results may not be significant. These results indicate that HMB oral supplementation can improve the physical function.

#### Subgroup analyses

To elucidate the effects of health status, age, gender, BMI, supplementary frequency, combination with exercise and combination with other substances on the efficacy of HMB oral supplementation, we conducted subgroup analysis on the above parameters. The results showed that health status, gender, and BMI had no significant impact on the effect of HMB, while age, HMB supplemental frequency, and whether combined with exercise intervention would affect the supplemental effect of HMB. For LM and five-time chair stand test, the results showed that HMB supplementation had an effect on people over 50 years old, but the effect was more prominent in the population over 65 years old. For LM, handgrip strength and gait speed, the supplementation once a day is the most effective, and combined exercise intervention has a better effect.

In addition, to further investigate the effects of HMB oral supplementation dosage, duration and relationship of dosage-duration on muscle mass, strength, and physical function, subgroup analyses were performed based on HMB dosage, duration and relationship of dosage-duration. The findings revealed that for LM, handgrip strength, and gait speed, a dosage of 3 g/day was found to have a significantly greater effect than a dosage of < 3 g/day. Similarly, for LM, handgrip strength, a supplementation duration > 12 weeks was found to have a significantly greater effect than a duration ≤ 12 weeks. And 3 g/day and > 12 weeks is the optimal dosage-duration response relationship. All of the above results are summarized in Table 2.

**TABLE 2:** Subgroup analysis of the effect of HMB on research indicators.

Subgroup	ASMM				LM				Handgrip strength			
	**Number of studies**	**WMD**	**95%CI**	**I^2^(%)**	**Number of studies**	**WMD**	**95%*CI***	**I^2^(%)**	**Number of studies**	**WMD**	**95%CI**	**I^2^(%)**
Dosage												
<3 g/d	4	1.480	–0.568, 3.528	96.40	4	0.221	0.011, 0.430	<0.001	3	0.962	–0.187, 2.111	69.66
3 g/d	2	1.716	–0.988, 4.419	94.44	11	0.292	0.134, 0.451	< 0.001	12	0.428	0.026, 0.829	17.51
Duration												
≤12 weeks	3	0.628	–0.342, 1.598	35.23	11	0.288	–0.032, 0.609	< 0.001	8	0.526	–0.177, 1.229	< 0.001
>12 weeks	3	1.12	–0.324, 2.564	84.12	5	0.295	0.097, 0.492	< 0.001	7	1.024	0.098, 1.951	38.7
Dosage-duration												
3g and > 12W	–	–	–	–	4	0.379	0.091, 0.667	< 0.001	2	2.406	0.700, 4.112	46.72
3g and ≤ 12W	2	1.362	0.084, 2.639	17.29	7	0.204	–0.119, 0.528	< 0.001	6	0.056	–0.865, 0.976	< 0.001
<3g and > 12W	3	1.12	–0.324, 2.564	84.12	1	0.204	–0.119, 0.528	< 0.001	5	0.208	–0.570, 0.985	< 0.001
<3g and ≤ 12W	1	–0.043	–0.917, 0.831	< 0.001	3	0.279	0.010, 0.548	< 0.001	2	1.187	0.097, 2.277	< 0.001
Health status												
Healthy	–	–	–	–	10	0.289	0.042, 0.536	19.74	4	0.720	–0.148, 1.588	< 0.001
Diseased	–	–	–	–	6	0.302	0.136, 0.467	< 0.001	11	0.525	–0.071, 1.121	64.42
Age												
< 65	–	–	–	–	3	0.225	0.156, 0.294	<0.001	3	3.313	–0.436, 7.063	58.16
≥ 65	–	–	–	–	13	0.285	0.161, 0.410	< 0.001	12	0.367	–0.056, 0.791	44.27
Gender												
M/F	–	–	–	–	11	0.275	0.068, 0.482	1.35	10	0.063	–0.426, 0.553	< 0.001
F	–	–	–	–	5	0.291	0.133, 0.449	< 0.001	4	0.626	0.235, 1.018	19.76
M	–	–	–	–	–	–	–	–	1	6.600	2.960, 10.240	< 0.001
BMI												
< 25	4	1.304	–0.442, 3.050	95.23	1	0.300	0.126, 0.474	<0.001	6	0.209	–0.334, 0.753	64.15
≥ 25 and < 30	1	0.300	–0.791, 1.31	< 0.001	10	0.193	–0.029, 0.414	< 0.001	6	1.230	–0.435, 2.894	57.5
Frequency												
One	–	–	–	–	5	0.318	0.164, 0.472	< 0.001	6	0.637	0.248, 1.025	< 0.001
Two	–	–	–	–	10	0.193	–0.021, 0.406	< 0.001	9	0.448	–0.339, 1.234	65.44
Three	–	–	–	–	1	1.000	–0.121, 2.121	< 0.001	0	–	–	–
Combination with exercise												
N	–	–	–	–	11	–0.053	–0.409, 0.304	< 0.001	11	0.526	–0.108, 1.160	64.85
Y	–	–	–	–	5	0.33	0.198, 0.463	< 0.001	4	0.769	0.160, 1.379	< 0.001
Combination with other substances												
Y	–	–	–	–	7	0.252	0.122, 0.382	< 0.001	1	0.46	–0.048, 0.967	55.43
N	–	–	–	–	9	0.632	0.203, 1.061	< 0.001	14	1.7	0.025, 3.375	< 0.001
	**Five-time chair stand test**				**Gait speed**				**6MWT**			
Dosage												
< 3 g/d	1	–0.500	–1.161, 0.161	<0.001	4	0.050	–0.054, 0.154	<0.001	1	7.280	–0.667,15.227	<0.001
3 g/d	3	–1.179	–2.401, 0.044	46.69	1	0.051	0.006, 0.097	17.14	2	8.368	–60.563,77.299	67.65
Duration												
≤ 12 weeks	–	–	–	–	–	–	–	–	–	–	–	–
> 12 weeks	–	–	–	–	–	–	–	–	–	–	–	–
Dosage–duration												
3g and > 12W	–	–	–	–	–	–	–	–	–	–	–	–
3g and ≤ 12W	3	–1.747	–3.035, –0.458	11.89	1	0.082	–0.026, 0.191	< 0.001	2	31.111	–16.678, 78.899	< 0.001
<3g and > 12W	–	–	–	–	–	–	–	–	–	–	–	–
< 3g and ≤ 12W	1	–0.5	–1.161, 0.161	<0.001	4	0.051	0.006, 0.097	17.14	1	7.28	–0.667, 15.227	<0.001
Health condition												
Healthy	–	–	–	–	–	–	–	–	1	7.280	–0.667, 15.227	< 0.001
Diseased	–	–	–	–	–	–	–	–	2	8.368	–60.563, 77.299	67.65
Age												
< 65	1	–0.375	–0.615, –0.135	<0.001	–	–	–	–	2	8.368	–60.563, 77.299	67.65
≥ 65	3	–0.618	–1.213, –0.023	24.59	–	–	–	–	1	7.280	–0.667, 15.227	< 0.001
Gender												
M/F	3	–1.179	–2.401, 0.044	46.69	4	0.050	–0.054, 0.154	17.14	1	36.500	4.301, 68.699	< 0.001
F	1	–0.500	–1.161, 0.161	< 0.001	1	0.051	0.006, 0.097	< 0.001	1	7.280	–0.667, 15.227	< 0.001
M	–	–	–	–	–	–	–	–	1	–35.600	–109.240, 38.040	< 0.001
BMI												
<25	2	–0.470	–0.970, 0.030	<0.001	–	–	–	–	–	–	–	–
≥25 and <30	1	–2.000	–4.273, 0.273	< 0.001	–	–	–	–	–	–	–	–
Frequency												
One	2	–0.840	–2.070, 0.391	35.2	4	0.051	0.006, 0.097	17.14	2	17.553	–9.791, 44.898	66.46
Two	2	–1.023	–2.574, 0.529	60.66	1	0.050	–0.054, 0.154	< 0.001	1	–35.600	–109.240,38.040	< 0.001
Three	–	–	–	–	–	–	–	–	–	–	–	–
Combination with exercise												
N	2	–0.835	–2.182, 0.511	39.29	2	0.023	–0.035, 0.080	< 0.001	–	–	–	–
Y	2	–1.035	–2.501, 0.430	58.63	3	0.075	0.024, 0.127	< 0.001	–	–	–	–
Combination with other substances												
Y	–	–	–	–	–	–	–	–	–	–	–	–
N	–	–	–	–	–	–	–	–	–	–	–	–

### Risk of bias analysis

**FIGURE 10:**
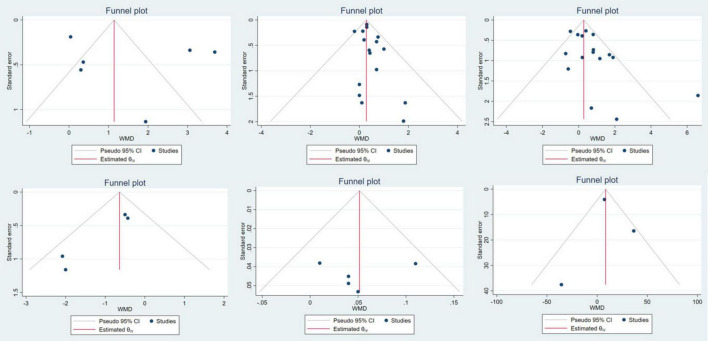
ASMM, LM, handgrip strength, five-time chair stand test, gait speed, and 6MWT bias funnel plot.

**FIGURE 11:**
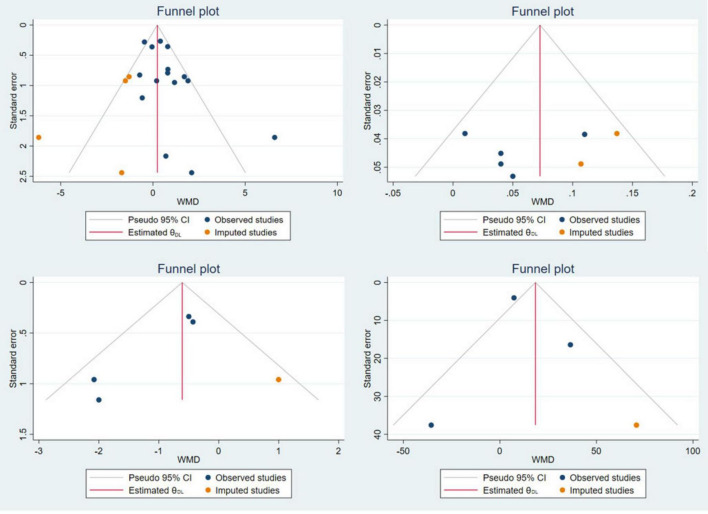
Handgrip strength, gait speed, five-time chair stand test, and 6MWT virtual pruning funnel plot.

In this study, funnel plot was used to evaluate risk of bias (Figure 10). And the symmetry of the funnel plot was further tested by Egger methods. The results were ASMM (*P* = 0.946), LM (*P* = 0.302), gait speed (*P* = 0.781), 6MWT (*P* = 0.523), handgrip strength (*P* = 0.022) and five-time chair stand test (*P* = 0.050). Because the funnel plot of gait speed and 6MWT is visually asymmetric, we conducted a virtual pruning analysis of the four indicators of gait speed, 6MWT, handgrip strength and five-time chair stand test. The results were gait speed (observed: WMD = 0.052 kg, 95% CI: 0.013 stand kg; observed + imputed: WMD = 0.073 kg, 95% CI: 0.035ed and kg), 6MWT (observed: WMD = 12.076 m, 95% CI: –13.8826 m, 95 kg; observed + imputed: WMD = 18.385 m, 95% CI: 5 m, 95 and t kg), handgrip strength (observed: WMD = 0.539 kg, 95% CI: 0.036rip st kg; observed + imputed: WMD = 0.237 kg, 95% CI: 5edrved str kg) and five-time chair stand test (observed: WMD = –0.730 kg, 95% CI: 5hair stand te kg; observed + imputed: WMD = –0.610 kg, 95% CI: 5edrved and kg) (Figure 11).

Because of a perceived insufficient number of included studies in ASMM, gait speed, five-time chair stand test and 6MWT, the test efficiency is low, and the results of funnel plot are difficult to assess the risk of bias. Therefore, we did not conduct too much analysis on these indicators, and the corresponding results need to be treated with caution. There is a significant risk of bias in handgrip strength. There are many reasons for the risk of bias, and the main sources may be heterogeneity between studies and publication bias. From the funnel plot, it is clear that two studies (29, 49) exhibit significant heterogeneity, which may be one of the factors contributing to the risk of bias. Secondly, positive, significant results are more likely to be published than negative and insignificant results, which may introduce bias into the overall research findings. It can be seen from the funnel plot that the positive results are significantly more than the negative results, indicating that publication bias may also be a source of bias risk for this indicator.

### GRADE assessment

The level of evidence of each outcome indicator included in the meta-analysis was evaluated. The results showed that LM, gait speed and five-time chair stand test are moderate quality evidence, ASMM is low quality evidence, handgrip strength and 6MWT are very low quality evidence. Table 3 contains the GRADE profile for the degree of certainty of the evidence.

**TABLE 3:** GRADE approach summary of findings and quality of evidence assessment.

Outcome	Certainty assessment							Sample volume		95% CI	Quality of evidence
	**No of studies**	**Design**	**Risk of bias**	**Inconsistency**	**Indirectness**	**Imprecision**	**Publication bias**	**Intervention control**	**Intervention control**		
ASMM	6	RCTs	Serious^a^	Serious^b^	No serious	No serious	No serious	378	375	0.03, 3.09	Low
LM	16	RCTs	Serious^a^	No serious	No serious	No serious	No serious	369	368	0.16, 0.41	Moderate
Handgrip strength	14	RCTs	Serious^a^	Serious^b^	No serious	No serious	Serious^c^	691	677	0.04, 1.04	Very low
Five–time chair stand test	4	RCTs	No serious	No serious	No serious	No serious	Serious^c^	97	97	1.35, 0.11	Moderate
Gait speed	5	RCTs	No serious	No serious	No serious	Serious^d^	No serious	164	168	0.01, 0.09	Moderate
6MWT	3	RCTs	Serious^a^	No serious	No serious	Serious^d^	Serious^c^	58	52	13.88, 38.03	Very low

CI, Confidence interval. Using the GRADE system, the quality of the evidence is broken down into four categories (high, moderate, low, and very low).

^a^The included study did not describe the blind method and the assignment hiding.

^b^I2 > 50%, there is significant heterogeneity.

^c^Funnel plot revealed evidence of bias.

^d^The sample size of the included studies was small, the confidence interval was wide, and the invalid line was crossed.

## Discussion

The previous meta-analysis explored the effect of HMB supplementation on muscle-related indicators in the population aged 65 and above, with less discussion on the supplementation dosage and duration. However, the age range of 50-65 is a critical period for preventing muscle loss. In this meta-analysis, we included studies on participants over the age of 50, and further investigated the effects of the supplementation dosage and duration, which are two key features of this study. The objective of this meta-analysis was to explore the effects of HMB oral supplementation on muscle mass, strength, physical function, and the dosage–duration response relationship, aiming to provide a scientific basis for the prevention and treatment of clinical sarcopenia. Data analyzed by summarizing all the RCTs meeting the inclusion criteria makes the results more comprehensive than using data from a single study, thereby enhancing the credibility of the findings. The results of the meta-analysis revealed that ASMM and LM were significantly higher in the intervention group than those of the control group, and the combined exercise intervention was more effective, indicating that HMB supplementation has a positive effect on muscle mass, and the combined exercise effect is better. These findings are consistent with those of previous studies. In this regard, Bear et al. (15) reported that HMB supplements have increased muscle mass across various clinical conditions. Wu et al. (50), in their meta-analysis, found that HMB supplementation is involved in preserving muscle mass among older adults and may be useful in the prevention of muscle atrophy. In addition, Molfino et al. (51) and Later et al. (22) concluded that HMB can mitigate exercise-induced muscle damage, further asserting that resistance training (RET) in conjunction with HMB supplementation significantly increases both muscle mass and strength. The underlying rationale for these results may be attributed to HMB’s capacity to inhibit protein breakdown, thereby reducing muscle degradation. In addition, HMB has been associated with increased endoplasmic reticulum calcium release, reduced fat content in skeletal muscle, increased oxygen metabolism, and the activation of satellite cells, consequently promoting muscle regeneration (16, 52, 53). We also considered the two indexes of muscle cross-sectional area and muscle volume. The results showed that HMB supplementation could significantly improve muscle cross-sectional area and muscle density (41, 46). However, because of the limited literature, we did not conduct a meta-analysis.

Several studies have investigated the impact of HMB on muscle strength. These supplements have been found to improve muscle strength in a variety of patients with decreased muscle mass and strength (15). In addition, meta-analyses have consistently demonstrated that HMB preparations can enhance upper and lower-limb muscle strength in the older adults (54, 55). Zhang et al. (56) and Zuo et al. (57) reported that combining HMB supplementation with RET can significantly boost lower-limb muscle strength in older individuals. Consistent with previous findings, the intervention group in our study exhibited significantly better results in handgrip strength and the five-time chair stand test than the control group, and the combined exercise intervention was more effective, indicating the positive impact of HMB oral supplementation on muscle strength, and it is best to combine exercise together. Contrary to these findings, a recent meta-analysis revealed that HMB supplementation had no substantial effect on muscle strength in adults aged 18–45 years (58). Our study, focused on adults > 50 years, contradicts this conclusion by revealing a positive regulatory effect of HMB supplementation on muscle strength in this age group. This reinforces the notion that the optimal population for HMB supplementation is middle-aged and older individuals. However, literature related to handgrip strength and the five-time chair stand test may have risk of bias, necessitating further research for confirmation in subsequent analyses.

Previous research has indicated that the combination of HMB and physical exercise can improve physical function. Li et al. (59) concluded that HMB combined with RET has a positive effect on physical function. In addition, a previous systematic review reported that HMB can improve physical function in older patients with sarcopenia or physical weakness (59). Consistent with these findings, our study also identified that HMB could improve physical function, with the experimental group demonstrating significantly better gait speed than the control group, especially in the case of combined intervention with exercise, the effect is more obvious. This reaffirms the beneficial effect of HMB supplementation on physical function. And this meta-analysis shows that the effect of HMB supplementation on 6MWT is not significant. This is inconsistent with the result of gait speed, although previous meta-analyses have found limited effects of HMB supplementation on physical function, such as Javier (55) found that HMB supplementation alone has limited or no effect on physical function in adults aged 50–80. But we should note that this result is only a combination of three studies, and the grading evaluation shows that 6MWT is very low quality evidence. And this is contrary to the previous study by Wu (60), who found that HMB combined with RET significantly affects the 6MWT of intensive care unit patients. We proposed three potential explanations for this discrepancy. Firstly, there may be a difference in intervention measures, since the RCTs we included in the 6MWT index were HMB supplementation alone, whereas Wu et al. study included RCTs of both HMB supplementation alone and HMB supplementation combined with physical exercise. Secondly, the research subjects are different. We did not limit the health status of the subjects, but Wu et al.’s were ICU patients, and the 6MWT index may be more suitable for evaluating this population. Thirdly, the 6MWT-related literature is very limited and grading assessment rated it as very low quality evidence. Based on comprehensive analysis, we believe that HMB supplementation has beneficial effects on physical function, but further research is needed to confirm these findings in the subsequent analysis.

Subgroup analysis showed that health status, gender and BMI had little effect on the supplemental effect of HMB. Although the results of LM, handgrip strength, gait speed and 6MWT indicate that gender and BMI have a slight impact on the effectiveness of HMB supplementation, the prominent results need further validation as the subgroups with prominent effects often involved only one RCT. After comprehensive analysis, we still believe that health status, gender and BMI are not the interfering factors of HMB supplemental effect. Age, supplementary frequency and whether combined with exercise intervention will affect the effectiveness of HMB. For LM and five-time chair stand test, HMB supplementation has an effect on the population over 50 years old, but the effect is more significant in the population over 65 years old. In this regard, we speculate that it may be caused by the disparity in sample size between subgroups. For LM, there were only two RCTs with subjects over 50 years and under 65 years, involving 67 subjects, and the other 670 subjects belonged to the subgroup of ≥ 65 years old. The gap in sample size between the two age groups may lead to the differences in the effect of HMB. Moreover, the five-time chair stand test may be more inclined to evaluate the elderly population, so it is expected that the effect of HMB supplementation will have a more prominent impact on people aged ≥ 65 years. For LM, handgrip strength and gait speed, supplement frequency once a day is more effective, and the effect of combined exercise intervention is better. Previous studies have also shown that HMB combined exercise can significantly improve muscle mass and strength (22, 51, 56, 57). Subgroup analysis also found that for LM, the effect of HMB oral supplements alone is more effective than that of combined with other substrates. Although the reason is not yet clear, we preliminarily believe that HMB supplementation alone may be more beneficial to LM.

The subgroup analysis revealed a significantly better effect of HMB supplementation at a dosage of 3 g/day than < 3 g/day. In this regard, Nissen et al. (21) studied the HMB supplementary dosage for the first time and found that a dosage of 3 g/day was more effective than 1.5 g/day. However, for participant safety, the majority of studies have maintained HMB dosage in the intervention group at ≤ 3 g/day, resulting in a notable gap in the investigation of dosage > 3 g/day. In this meta-analysis, only one study employed a supplemental dosage > 3 g/day, explicitly indicating that HMB supplementation could enhance muscle mass and physical function in elderly patients with hip fractures and prevent the occurrence of sarcopenia. At present, studies have shown that HMB is a relatively safe nutritional supplement. Animal experiments have shown no significant side effects of HMB, even at high dosages of 100 g/day for 4 days (16). Similarly, human studies demonstrated no side effects with dosages as high as 6 g/day for up to 1 month (16). Consequently, there is a compelling need for researchers to expand their investigations into the effects of supplementation with dosage of > 3 g/day. In addition, more recent studies are warranted to comprehensively evaluate HMB supplementation’s efficacy.

In addition, this meta-analysis revealed that an HMB supplementation duration > 12 weeks had a better effect than a duration ≤ 12 weeks. Several studies have demonstrated that supplementation duration ≤ 6 weeks show no significant difference between the control and placebo groups, whereas supplementation duration > 6 weeks positively impact muscle strength (19, 20, 61–63). Nissen et al. (21) and Wilson et al. (64) reported that HMB supplementation for > 2 weeks results in a more pronounced reduction in protein decomposition and skeletal muscle damage compared with supplementation for only 1 week. Slater and Jenkins (22) found that in the context of long-term endurance training, HMB can mitigate muscle protein degradation and muscle damage, resulting in increased muscle mass and strength, although its impact is less significant in short-term training. This is consistent with our research findings, suggesting that a longer supplementation duration may yield a better effect.

This meta-analysis conducted a subgroup analysis of the combined effects of dosage and duration. For LM and handgrip strength, there are complete data for the four subgroups. The analysis results showed that 3 g/d and > 12 weeks were the best, which is better than subgroup analysis based solely on dosage and duration. The results of ASMM and five-time chair stand test showed that the effect of subgroup 3 g/d and ≤ 12 weeks is significant, but we don’t have the relevant data of longer duration of this dosage, so we cannot confirm whether the effect of 3 g/d and > 12 weeks is more significant. For gait speed, the effect of < 3 g/d and ≤ 12 weeks is already significant, but due to the lack of data for 3 g/d and > 12 weeks, it cannot be confirmed whether the dosage-duration response relationship is more significant. LM and handgrip strength are two key indicators for evaluating muscle mass and strength, and the data for each subgroup is complete. Therefore, the subgroup analysis results of the dosage-duration response relationship between these two indicators are more reliable. In summary, we believe that 3 g/d and > 12 weeks may be the optimal dosage-duration response relationship for HMB supplementation.

This meta-analysis has several acknowledged advantages. First of all, compared to most previous meta-analyses, this meta-analysis added the study on the 50–65 age group and obtained positive research results. Secondly, this meta-analysis added subgroup analysis of dosage, duration, and dosage-duration response relationships, and concluded that HMB is suitable for high-dosage and long-duration supplementation. Thirdly, this meta-analysis conducted subgroup analysis and discussion on parameters that may affect muscle indicators, such as health status, age, gender, BMI, frequency, combination with exercise, and combination with other substances. Last but not least, in order to evaluate the degree of certainty of outcome evidence, this study used the GRADE method to assess the level of evidence.

However, when interpreting our research results, some limitations should be considered. First of all, there is little literature on participants between 50 and 65 years old. Only 4 studies are in this age group, and the rest are all over 65 years old (39, 42, 48, 49). This makes the meta-analysis of ASMM and gait speed only involved objects over 65 years old. And for five-time chair stand test, only one study was between 50 and 65 years old. The results of these three indicators may be more inclined to the elderly population. Even so, our analysis found a promising intervention effect of this age group on other indicators, such as LM and handgrip strength. These two indicators are important for directly evaluating muscle mass and strength. It is worth noting that GRADE assessment shows handgrip strength and 6MWT are very low quality evidence. The results of these two indicators should be further verified in the future. Secondly, five-time chair stand test may have some risk of bias and should be carefully considered. Thirdly, the included studies used various indicator measurement techniques that may provide different results, making it difficult to compare datasets. However, all RCTs used standard protocols and high-quality equipment, so we believe that the differences in measurement techniques between the studies may have a minor impact on the results. Fourthly, subgroup analysis of important parameters of some indicators cannot be performed due to the lack of research data. Therefore, it is necessary to supplement more high-quality RCTs in subsequent studies.

## Conclusion

Oral supplementation of HMB can improve muscle mass, strength and body function. Positive effects on muscle mass and strength have been observed in the population aged 50–65. We recommend to implement supplementation at a dosage of no less than 3 g for more than 12 weeks to achieve optimal benefits. This meta-analysis added the study of dosage, duration, and dosage-duration response relationships. The results of this study will provide a reference for the dosage and duration of clinical HMB supplement, and provide a basis for the prevention and treatment of clinical sarcopenia. However, oral supplementation of HMB may also have some potential effects in practical applications, such as whether it will cause gastrointestinal problems, whether it will increase the burden on the liver, and whether it has an effect on hormone levels, which still needs to be further verified in future studies. In addition, due to limited number of studies included and the risk of bias, more studies, especially high-quality RCTs, are needed to further evaluate or confirm the beneficial effects of HMB on muscle strength and physical function.

## Data Availability

The original contributions presented in the study are included in the article/Supplementary material, further inquiries can be directed to the corresponding authors.
