# Supplementary material for: Effects of oral supplementation of β -hydroxy-β -methylbutyrate on muscle mass and strength in individuals over the age of 50: a meta-analysis
Source: Front Nutr. 2025 Apr 3;12:1522287. doi: 10.3389/fnut.2025.1522287 (PMC12003145; doi:10.3389/fnut.2025.1522287)
Supplement: Supplementary file 1 [file Table_1.doc]

| **Database** | **Keywords** | **Records** |
| --- | --- | --- |
| PubMed | (((((((((beta hydroxy beta methyl butyrate) OR (hydroxy methyl butyrate)) OR (b-hydroxy-b-methyl butyrate)) OR (beta-hydroxy-beta-methylbutyrate)) OR (β-hydroxy-β-methylbutyrate)) OR (HMB-Ca)) OR (HMB)) OR (hmb)) AND (((((Aged) OR (elder)) OR (elderly)) OR (old adult)) OR (Middle Aged))) AND ((((((((((((((((((((Muscles) OR (muscle weakness)) OR (Muscular Atrophy)) OR (Muscular Atrophies)) OR (Muscle Atrophies)) OR (Muscle Atrophy)) OR (amyotrophic)) OR (muscle loss)) OR (Muscle wasting)) OR (Sarcopenia)) OR (Sarcopenias)) OR (muscle mass)) OR (lean mass)) OR (fat free mass)) OR (muscle volume)) OR (muscle cross sectional area)) OR (six-minute Walking)) OR (Walking)) OR (walk distance)) OR (five time chair stand test)) | 455 |
| Cochrane Library | #(beta hydroxy beta methyl butyrate) OR (b-hydroxy-b-methyl butyrate) OR (beta-hydroxy-beta-methylbutyrate) OR (β-hydroxy-β-methylbutyrate) OR (HMB) and #(Aged) OR (elder) OR (elderly) OR (old adult) OR (Middle Aged) and #(muscle weakness) OR (Muscular Atrophy) OR (Sarcopenia) OR (muscle mass) OR (amyotrophic) | 119 |
| Web of Science | beta hydroxy beta methyl butyrate (Topic) or hydroxy methyl butyrate (Topic) or b-hydroxy-b-methyl butyrate (Topic) or beta-hydroxy-beta-methylbutyrate (Topic) or β-hydroxy-β-methylbutyrate (Topic) or HMB-Ca (Topic) or HMB (Topic) and hmb (Topic) and Preprint Citation Index (Exclude – Database) AND Aged (Topic) or elder (Topic) or elderly (Topic) or old adult (Topic) or Middle Aged (Topic) and Preprint Citation Index (Exclude – Database) AND Muscles (Topic) or muscle weakness (Topic) or Muscular Atrophy (Topic) or Muscular Atrophies (Topic) or Muscle Atrophies (Topic) or Muscle Atrophy (Topic) or amyotrophic (Topic) or muscle loss (Topic) or Muscle wasting (Topic) or Sarcopenia (Topic) or Sarcopenias (Topic) or muscle mass (Topic) or lean mass (Topic) or fat free mass (Topic) or muscle volume (Topic) or muscle cross sectional area (Topic) or six-minute Walking (Topic) or Walking (Topic) or walk distance (Topic) or five time chair stand test (Topic) and Preprint Citation Index (Exclude – Database) | 811 |
| ScienceDirect | (b-hydroxy-b-methyl butyrate) OR (beta-hydroxy-beta-methylbutyrate) OR (β-hydroxy-β-methylbutyrate) OR (HMB) and (Aged) OR (elderly) OR (old adult) OR (Middle Aged) and (muscle weakness) OR (Muscular Atrophy) OR (Sarcopenia) | 1249 |
| EBSCO | TX ( beta hydroxy beta methyl butyrate or hydroxy methyl butyrate or b-hydroxy-b-methyl butyrate or beta-hydroxy-beta-methylbutyrate or β-hydroxy-β-methylbutyrate or HMB-Ca or HMB or hmb ) AND TX ( Aged or elder or elderly or old adult or Middle Aged ) AND TX ( Muscles or muscle weakness or Muscular Atrophy or Muscular Atrophies or Muscle Atrophies or Muscle Atrophy or amyotrophic or muscle loss or Muscle wasting or Sarcopenia or Sarcopenias or muscle mass or lean mass or fat free mass or muscle volume or muscle cross sectional area or six-minute Walking or Walking or walk distance or five time chair stand test ) | 15 |
| China Journal Full-Text Database (CNKI)* | (Topic: beta hydroxy beta methyl butyrate) OR (Topic: β-hydroxy-β-methylbutyrate) OR (Topic:calcium β-hydroxy-β-methylbutyrate hydrogels) OR (Topic: HMB) OR (Topic: Ca-HMB) OR (Topic: HMB-Ca) AND (Topic: Aged) OR (Topic: Elderly group) OR (Topic: over the age of 65) OR (Topic: over the age of 60) OR (Topic: community elderly) OR (Topic: over the age of 50) OR (Topic: Middle Aged) AND (Topic: old adult) OR (Topic: Sarcopenia) OR (Topic: Muscle wasting) OR (Topic:muscle loss) OR (Topic: muscle weakness) OR (Topic: muscle mass) OR (Topic: muscle strength) OR (Topic: muscle volume) OR (Topic: muscle cross sectional area) OR (Topic: skeletal muscle) | 10 |
| Wan Fang* | All Fields: (beta hydroxy beta methyl butyrate or β-hydroxy-β-methylbutyrate or calcium β-hydroxy-β-methylbutyrate hydrogels or HMB or Ca-HMB or HMB-Ca) and All Fields: (Aged or Elderly group or over the age of 65 or over the age of 60 or community elderly or over the age of 50 or Middle Aged) and All Fields: (Sarcopenia or Muscle wasting or muscle loss or muscle weakness or muscle mass or muscle strength or muscle volume or muscle cross sectional area or skeletal muscle) | 12 |

* represents the Chinese database, using the corresponding Chinese search terms to search.
